# A New Wound-Healing Tool Based on *Glycyrrhiza glabra* Extract-Loaded Ufasomes on Spanish Broom Dressings

**DOI:** 10.3390/molecules29163811

**Published:** 2024-08-11

**Authors:** Simone Rossello, Manuela Mandrone, Teresa Cerchiara, Ilaria Chiocchio, Martina Rossi, Fabio Chinnici, Valentina Sallustio, Maria Aponte, Giuseppe Blaiotta, Barbara Luppi, Angela Abruzzo, Federica Bigucci, Concettina Cappadone

**Affiliations:** 1 Drug Delivery Research Laboratory, Department of Pharmacy and Biotechnology, Alma Mater Studiorum, University of Bologna, Via San Donato 19/2, 40127 Bologna, Italy; simone.rossello2@unibo.it (S.R.); valentina.sallustio2@unibo.it (V.S.); angela.abruzzo2@unibo.it (A.A.); federica.bigucci@unibo.it (F.B.); 2 Pharmaceutical Botany Laboratory, Department of Pharmacy and Biotechnology, Alma Mater Studiorum, University of Bologna, Via Irnerio 42, 40127 Bologna, Italy; manuela.mandrone2@unibo.it (M.M.); ilaria.chiocchio2@unibo.it (I.C.); 3 Pharmaceutical Biochemistry Laboratory, Department of Pharmacy and Biotechnology, Alma Mater Studiorum, University of Bologna, Via San Donato 19/2, 40127 Bologna, Italy; martina.rossi12@unibo.it (M.R.); concettina.cappadone@unibo.it (C.C.); 4 Department of Agricultural and Food Sciences, Alma Mater Studiorum, University of Bologna, Viale Fanin 40, 40127 Bologna, Italy; fabio.chinnici@unibo.it; 5 Department of Agricultural Sciences, University of Naples “Federico II”, 80055 Portici, Italy; aponte@unina.it (M.A.); blaiotta@unina.it (G.B.)

**Keywords:** *Glycyrrhiza glabra* L., extract encapsulation, ufasomes, Spanish broom, wound dressings, WS1 fibroblast, wound healing, antioxidant activity

## Abstract

The development of innovative products for restoring skin integrity and promoting wound healing is still a challenge. The aim of this work was to evaluate an innovative Spanish broom wound dressing impregnated with *Glycyrrhiza glabra* extract-loaded ufasomes to improve wound healing. Ufasomes were characterized in terms of size, polydispersity index, entrapment efficiency, zeta potential, and stability. In addition, in vitro release studies and biocompatibility, biosafety, and scratch tests on WS1 fibroblasts were performed. The loaded ufasomes showed a nanometric size (<250 nm), good size distribution (lower than 0.3), and appropriate encapsulation efficiency (~67%). Moreover, the lipid vesicles showed good stability during the storage period and allowed for a slow release of glycyrrhizin, the main bioactive compound of the extract. Biological studies revealed that loaded vesicles are not cytotoxic, are hemocompatible, and lead to the complete closure of the scratch after about 33 h. To conclude, the results suggest that the developed dressings can be efficiently used to promote the healing process.

## 1. Introduction

The global wound dressing market is increasing and is predicted to rise at a compound annual growth rate (CAGR) of 4.16% from 2024 to 2030 [1]. Currently, researchers are paying continuous attention to exploring advanced wound care technologies and product enhancement and the development of innovative wound dressings to accelerate the healing. Moreover, it is worth noting that some wound dressings may not be suitable for all types and stages of wounds. A wound is defined as any damage to skin integrity due to incisions, burns, scalds, and human lesions (diabetic foot, venous ulcers, etc.) [2]. In recent years, plant-based extracts like *Glycyrrhiza glabra* L., *Achillea millefolium* L., *Aloe vera* L., and *Calendula officinalis* L. have been proposed as alternative to medicinal compounds for improving the healing process [2]. *Glycyrrhiza glabra* L., a perennial herbaceous plant of the *Fabaceae* family, grows spontaneously in Italy and specifically in Calabria and Abruzzo. It is known that licorice root extract contains many biologically active compounds such as triterpene saponins, glycyrrhizin, flavonoids, and isoflavonoids, which are mainly responsible for antioxidant, anti-inflammatory, and antimicrobial activity [3,4]. Unfortunately, *Glycyrrhiza glabra* L. extract is characterized by sensitivity to oxidation and photolysis, which decreases its biological activity. Generally, to preserve biological properties, the encapsulation of natural extracts into nanosystems is a strategy that has been investigated [5,6]. In this regard, for the first time, our research group investigated the encapsulation of *Glycyrrhiza glabra* extract (GG root extract) into ufasomes and their use in developing Spanish broom wound dressings by impregnation.

Ufasomes are “unsaturated fatty acid vesicles” that were discovered for the first time by Gebicki and Hicks in 1973 [7]. Typically, oleic acid and linoleic acid are the main components of ufasomes, and, compared with liposomes, they present many advantages such as good biocompatibility, the easy bioavailability of raw material, and a simple preparation method. To accelerate the healing process, ufasomes have been used in conjunction with textiles, which are still the most used material for the treatment of wounds [5]. Among available textiles, we proposed Spanish broom dressings thanks to the availability, renewability, and cleaner and more resilient cultivation [8]. Our previous studies have already investigated the possibility of Spanish broom dressings being used to deliver various bioactive compounds in the treatment of skin wounds. To the best of our knowledge, no investigation has been conducted on GG root extract-loaded ufasomes intended for wound healing.

In this work, ufasomes (UFAs) made up of oleic and linoleic acid and ufasomes based only on oleic acid, which we indicated as aosomes (AOs), were prepared, respectively. Lipid vesicles were characterized for their physico-chemical and functional properties, such as their size, particle size distribution, ζ potential, encapsulation efficiency, stability over time, and ability to release glycyrrhizin (GZ). In addition, lipid vesicle cytotoxicity and cell proliferation activity were evaluated through a 3-(4,5-dimethylthiazol-2-yl)-2,5-diphenyl tetrazolium bromide (MTT) assay and cell cycle analysis on WS1 fibroblasts, respectively. The vesicles’ biosafety and antioxidant activity were also evaluated. Finally, the loaded vesicles were sunk into Spanish broom wound dressings, and a scratch test was performed to assess the wound-healing properties.

## 2. Results and Discussion

### 2.1. Chemical Analysis of GG Root Extract

The selection of the appropriate solvent is the first step for successfully isolating bioactive compounds from plants. Generally, the solvents accepted for use in the pharmaceutical industry are water, ethanol, and glycerol [9]. In this work, we selected a mixture of ethanol/water 50:50 *v*/*v* to extract the highest content of flavonoids and polyphenols that, along with the main bioactive compound of *Glycyrrhiza glabra* L. roots, GZ, are known to possess antioxidant and anti-inflammatory activity, respectively [4]. The content of GZ in the root extract was determined by HPLC and resulted to be 86.7 µg/mg of the extract. The total phenolic content (TPC) and the total flavonoid content (TFC) were 78.7± 0.21 µg GAE/mg of the extract and 7.6 ± 0.3 µg QE/mg of the extract, respectively. The antioxidant activity (AA%), measured by the DPPH test, was 81.16 ± 0.69%, confirming the high antioxidant activity of the GG root extract. It is worth mentioning that the extract chemical composition is deeply influenced by agro-climatic conditions such as the temperature, rainfall, altitude, humidity, or soil composition at which the root grows [10]. Our findings showed that GG root extract is rich in GZ and, more generally, in phenolic compounds.

### 2.2. Antibacterial Activity of GG Root Extract

**Figure 1:**
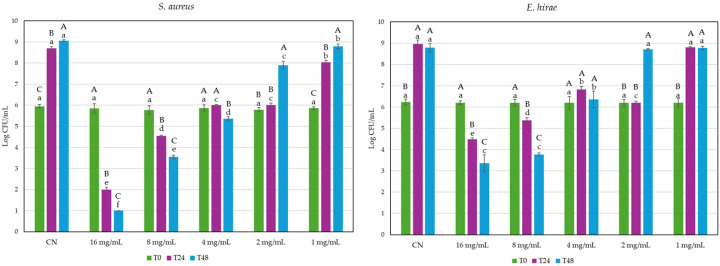
Effect of five different concentrations of GG root extract on *S. aureus* DSM799 and *E*. *hirae* DSM3320 over 48 h using TSB medium. Significant differences among data were computed by using ANOVA and Tukey *t*-tests (*p* < 0.05) (XL Stat 2012.6.02 statistical pocket, Addinsoft Corp., Paris, France). Lowercase: different samples at the same incubation time. Uppercase: the same sample at different incubation times.

According to the agar well diffusion assay, the extract proved to be active against *S. aureus* DSM799 and *E. hirae* DSM3320 (Figure 1), but not against *Escherichia coli* DSM11250 (Figure S1: Growth inhibition in the presence of different concentrations of GG root extract). *Glycyrrhiza glabra* extract has been proven to be uniquely effective against Gram-positive bacteria by Sallustio et al. [11]. Specifically, 4 mg/mL was the ultimate concentration capable of promoting the appearance of a distinct inhibition halo of about 0.2 mm in both strains.

Conversely, according to the time–kill assay, the extract’s impact on the two indicators was rather different. At a concentration of 16 mg/mL, the extract was able to lower the initial microbial population (10^6^ CFU/mL) of *S. aureus* by about 4 Log CFU/mL in 24 h and by approximately 5 Logs in 48 h at a dosage of 16 mg/mL. At the same concentration, the *E. hirae* strain appeared to be less affected: roughly, 1.7 Logs reduction in 24 h and 2.85 Logs reduction in 48 h.

Antibacterial activity was interpreted as bactericidal when a reduction of 3 Log CFU/mL (99.9% kill) occurred, compared with the initial inoculum at time 0, and bacteriostatic if the inoculum was reduced by between 0 and 3 Log CFU/mL [12,13]. In *S. aureus*, the GG root extract is bactericidal only for concentrations in the range 16–8 mg/mL; in 24 and 48 h, the reduction was greater than 99.9% and 99.99%, respectively.

### 2.3. Encapsulation of GG Root Extract Into Vesicles and Their Characterization

The GG root extract was encapsulated into lipid vesicles, namely, UFAs and AOs, respectively. UFAs were prepared using both of the unsaturated fatty acids, oleic and linoleic acid, while AOs were obtained using oleic acid alone. The unsaturated fatty acids have anti-inflammatory action that may promote the wound-healing process [14], rendering these vesicles promising nanocarriers capable of delivering the GG root extract for the treatment of wounds. Additionally, fundamental physico-chemical parameters such as the mean size, polydispersity index, and zeta potential must be assessed to characterize colloidal systems. As can be seen in Table 1, unloaded and loaded fatty vesicles have nanometric sizes of less than 250 nm. UFAs and AOs loaded with GG root extract had smaller sizes (~158 nm) than empty UFAs and AOs (~219 nm). This effect is due to the different interactions between the extract compounds (saponins, flavonoids, triterpenes, and sterols) and the vesicle components. Regarding the PDI, all samples prepared showed low PDI values, indicating the good homogeneity of the vesicles. Moreover, the zeta potential values of the vesicles were negative, and a zeta potential value of ±30 mV indicates that the vesicles tend to repulse each other, thus favoring system stability over time.

The encapsulation efficiency (EE%) of the GG root extract into UFAs and AOs was similar, with no statistical differences between the two types of vesicles (∼67%, Table 1), probably due to GZ saturation in the lipid bilayers. To our knowledge, this is the first work on the encapsulation of natural extract into UFAs and AOs.

### 2.4. Stability Studies

**Figure 2:**
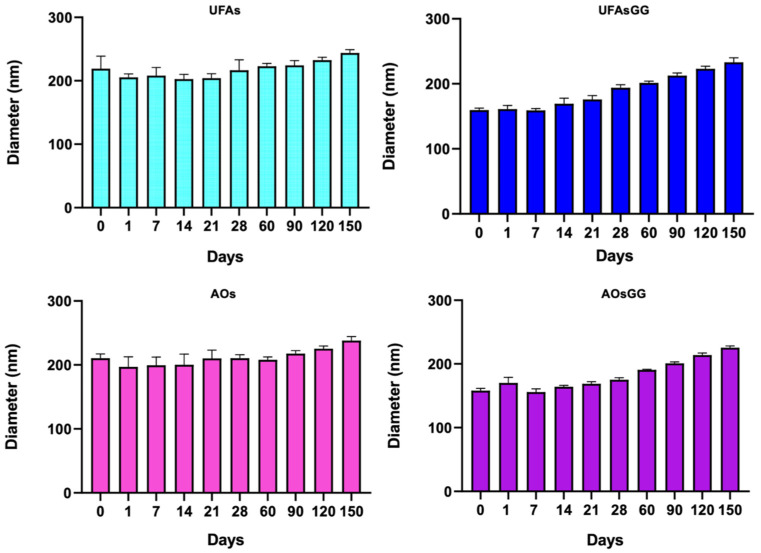
Sizes of unloaded and loaded UFAs and AOs during five months of storage at 4.0 ± 1.0 °C (data expressed as the mean ± SD, n = 3).

The physical and chemical integrity of any pharmaceutical product must be guaranteed during its shelf life [15]. Therefore, stability studies represent a crucial step in the development of new formulations, including nanovesicles that are undeniably considered moderately unstable colloidal systems. In this work, stability studies were performed to observe the physical changes undergone by the vesicles over a period of 150 days at 4 °C. Figure 2 shows the variation in the size of the unloaded and loaded UFAs as well as the AOs over a storage period of 5 months at 4.0 ± 1.0 °C. No relevant changes in the size of the unloaded UFAs and AOs were observed over this period; after 3 months, a slight increase in size (*p* < 0.01) was observed for both of the loaded formulations.

**Figure 3:**
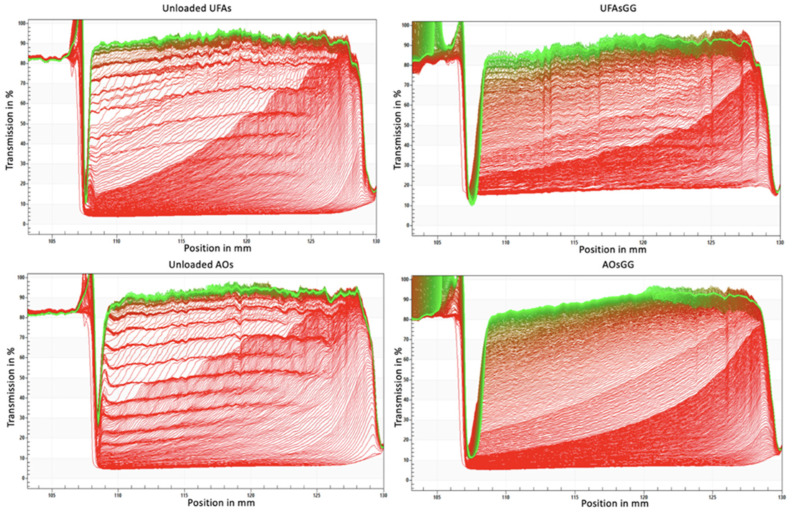
LUMiSizer^®^ profiles with an analysis of instability phenomena observed for unloaded and loaded UFAs and AOs.

To optimize and ensure the stability of UFAs, it is crucial to analyze dispersion–separation processes such as creaming, sedimentation, flocculation, coalescence, or phase separation [16]. In this work, stability studies of UFAs and AOs were performed, recording the transmission profiles after 24 h of storage time, using the analytical photocentrifuge LUMiSizer^®^ (L.U.M. GmbH, Berlin, Germany). Accelerated stability studies revealed that all the formulations show, as a separation profile, a creaming effect over time (Figure 3). This effect is slower for the loaded vesicles than for the unloaded ones.

**Figure 4:**
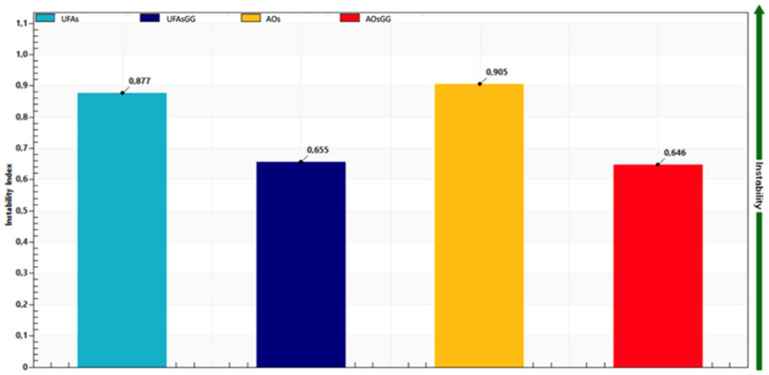
Instability index of unloaded and loaded UFAs and AOs after approx. 5 months.

Additionally, the LUMiSizer^®^ offers the acquisition of quantitative data—specifically, the instability index (I.I.). This parameter can be computed based on the clarification—more precisely, the increase in transmission due to phase separation by sedimentation or creaming at a specified separation time, divided by the maximum clarification. The instability index (I.I.) is a dimensionless number, ranging from 0 (most stable) to 1 (most unstable). Therefore, samples with rapid clarification rates tend to exhibit greater instability for the same overall clarification [17]. As shown in Figure 4, loaded UFAs and loaded AOs have an I.I. of 0.655 and 0.646, respectively, while unloaded UFAs and AOs have an I.I. of 0.877 and 0.905, respectively. These results indicate that GG root extract-loaded UFAs and GG root extract-loaded AOs are more stable than the unloaded ones.

### 2.5. In Vitro Release Studies

The release study of GZ from the UFA, AOs, and free extract solution (1 mg/mL, GG *root extract*) was performed in a mixture of PBS:EtOH 7:3 *v*/*v* at a temperature of 32 ± 1 °C for 24 h.

**Figure 5:**
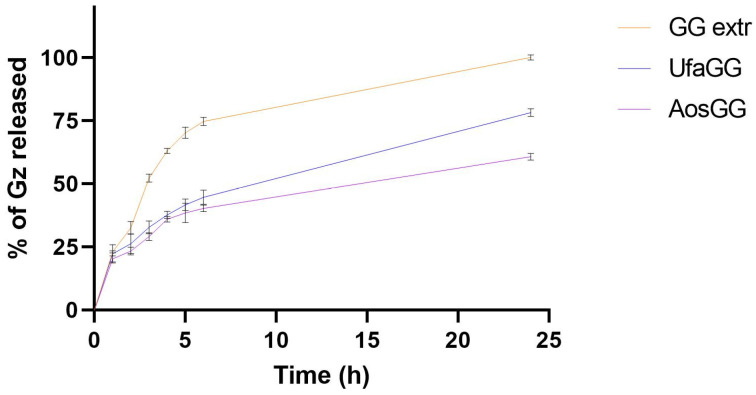
In vitro release of the GG root extract, loaded UFA, and AOs (data expressed as the mean ± SD, n = 3).

The percentage amount of GZ released over time is shown in Figure 5. The results showed that the free extract exhibited a release of 100.19 ± 1.03% of GZ after 24 h. On the other hand, the extract encapsulated into lipid vesicles is slowly released over time, achieving a release percentage of 78.28 ± 0.19% and 60.80 ± 1.34%, respectively, at 24 h (*p* < 0.05). This could be attributed to the ability of the nanovesicles to retain GZ in the lipid membrane.

### 2.6. Cell Viability Studies

**Figure 6:**
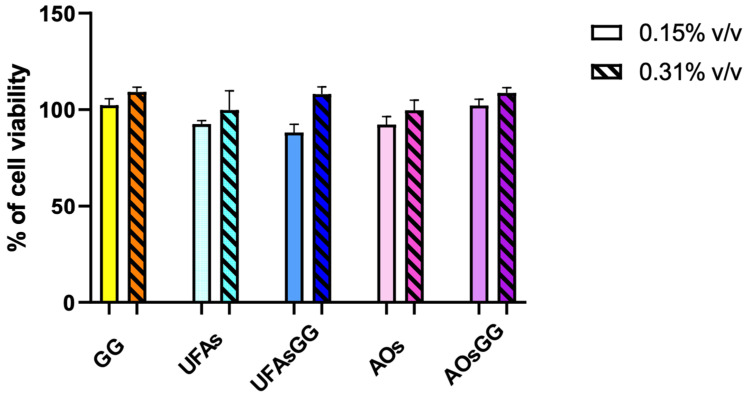
Viability in human fibroblast WS1 cells after treatment with different concentrations of lipid vesicles for 48 h. Data are reported as the average of three experiments ± SD, n = 3.

Pharmaceutical formulations for wound healing have to meet the essential criteria of biocompatibility and the absence of cytotoxicity towards the cell metabolism. To evaluate cell viability, the MTT assay was performed on WS1 human fibroblasts, which were chosen as the cell model, as they play a crucial role in the skin regeneration process. The treatments with the pure extract proved to be non-toxic at concentrations of 9.3 µg/mL and 18.6 µg/mL, corresponding to 0.15% and 0.31% *v*/*v*, respectively. Similarly, the unloaded and loaded UFAs as well as the AOs showed high biocompatibility at all tested concentrations. Indeed, no metabolic reduction was observed until 48 h (*p* > 0.05) (Figure 6).

### 2.7. Cell Cycle Evaluation by Flow Cytometry

**Figure 7:**
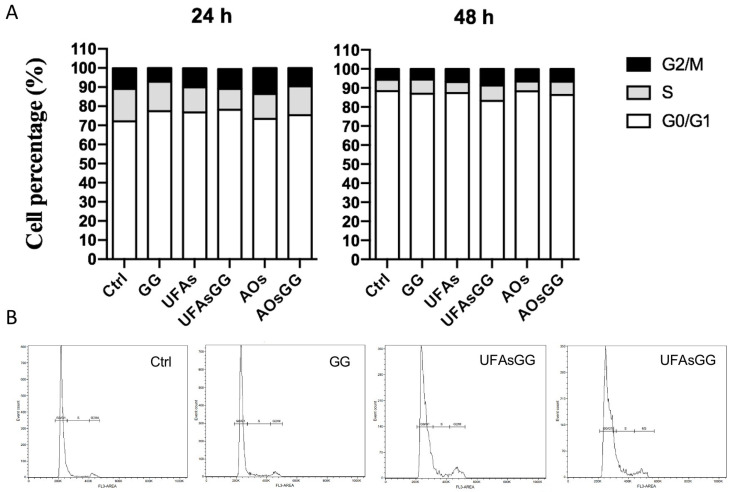
Cell cycle analyses. The cell cycle was evaluated after 24 or 48 h of treatment. (**A**) Percentage distribution of cells in the different cell cycle phases. (**B**) Cytograms showing the cell cycle profile of a representative experiment.

In order to better assess the absence of cytotoxic effects, the analysis of the cell cycle was performed by cytometry. The results showed that the percentage of the cell distribution is not altered by treatment with nanovesicles and/or extract, neither at 24 h nor 48 h (Figure 7). This further evidence confirmed the suitability of loaded UFAs and AOs for wound healing.

### 2.8. Biosafety

**Figure 8:**
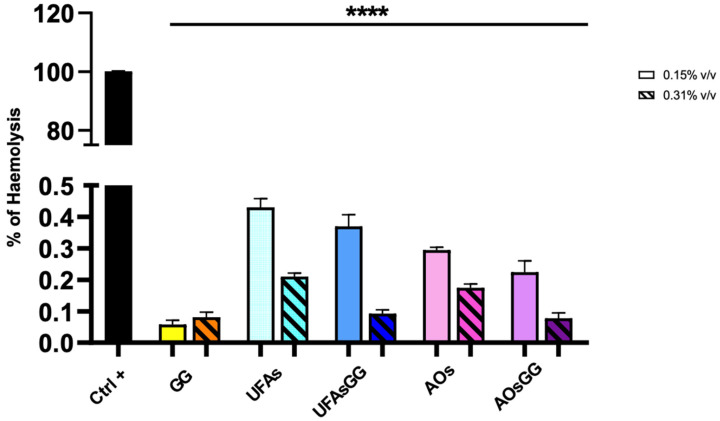
Hemolytic rate after treatment with GG root extract and loaded and unloaded UFAs and AOs (**** *p* < 0.0001 between all samples under the black line compared to Ctrl+).

Hemocompatibility is an important requirement of medical devices for their safe use in wound healing [18]. The national safety standards recommend that the hemolysis rate should be less than 5% to consider the formulation biosafe. The results showed that Glycyrrhiza glabra extract, unloaded and loaded UFAs, and AOs are biosafe, showing a hemolysis rate less than 0.5% (Figure 8).

### 2.9. Intracellular ROS Assay

**Figure 9:**
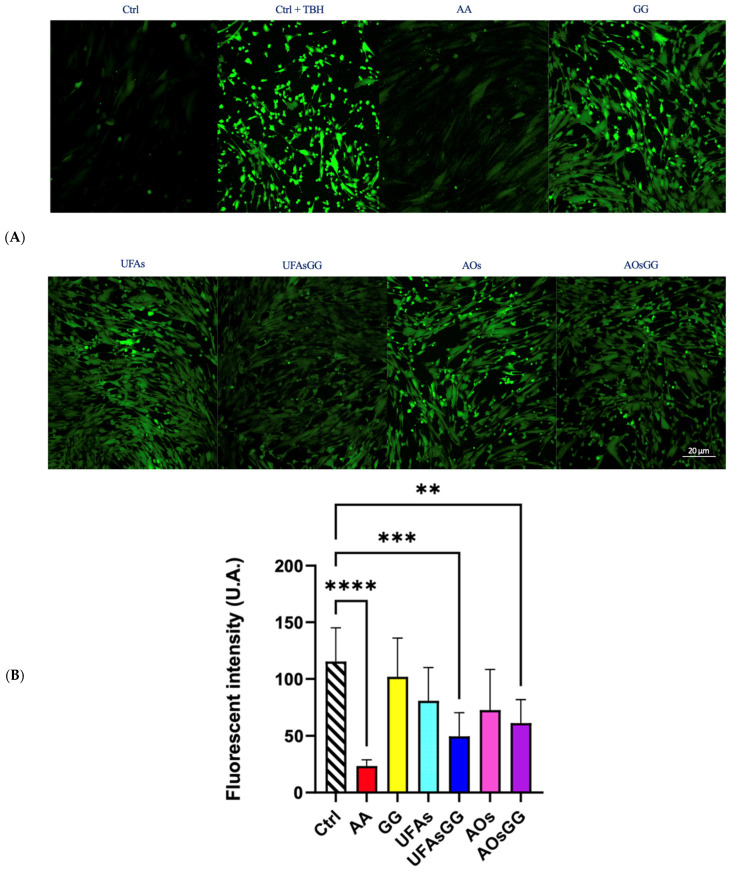
(**A**) Representative images of WS1 stained with H_2_DFCDA (green) after treatment with nanovesicles for 24 h. (**B**) Fluorescence intensity in WS1 fibroblasts after treatment with nanovesicles for 24 h. Data are expressed as the mean ± SD, n = 3 (** *p* < 0.01, *** < 0.001, **** < 0.0001).

To determine the antioxidant activity of the lipid vesicles, the intracellular ROS levels were quantified by confocal microscopy on fibroblasts, after oxidative stress induced by tert-butyl hydroperoxide (TBH) and H_2_DCFDA staining [19]. Figure 9A shows the images acquired, after 24 h of treatment, with all the formulations shown. It is evident that there was increased fluorescence after cell treatment with the oxidant TBH. In contrast, in the control samples or in the presence of the antioxidant ascorbic acid, the green fluorescence is almost absent, which corresponds to the basal concentrations of reactive oxygen species.

Interestingly, the nanoformulations with the GG root extract also reduce the fluorescence intensity. It is noteworthy that the pure extract is not effective, indicating the necessity to encapsulate it into protective nanovesicles.

The quantitative analyses of the fluorescence intensity are reported in Figure 9B. Our results demonstrate that GG root exxtract-loaded UFAs and GG root extract-loaded AOs are able to reduce the intracellular ROS levels by approximately 50% with respect to the positive control. We used the well-known antioxidant ascorbic acid as a negative control, which is already exploited in different pharmaceutical preparations for the healing of wounds [20]. Interestingly, both of the GG root extract-loaded nanovesicles significantly reduced the oxidative process, proving to be eligible for the wound dressings. Considering that increased ROS levels are believed to be responsible for wound chronicity, GG root extract-based formulations are even more interesting for wound care.

### 2.10. Wound-Healing Assay

**Figure 10:**
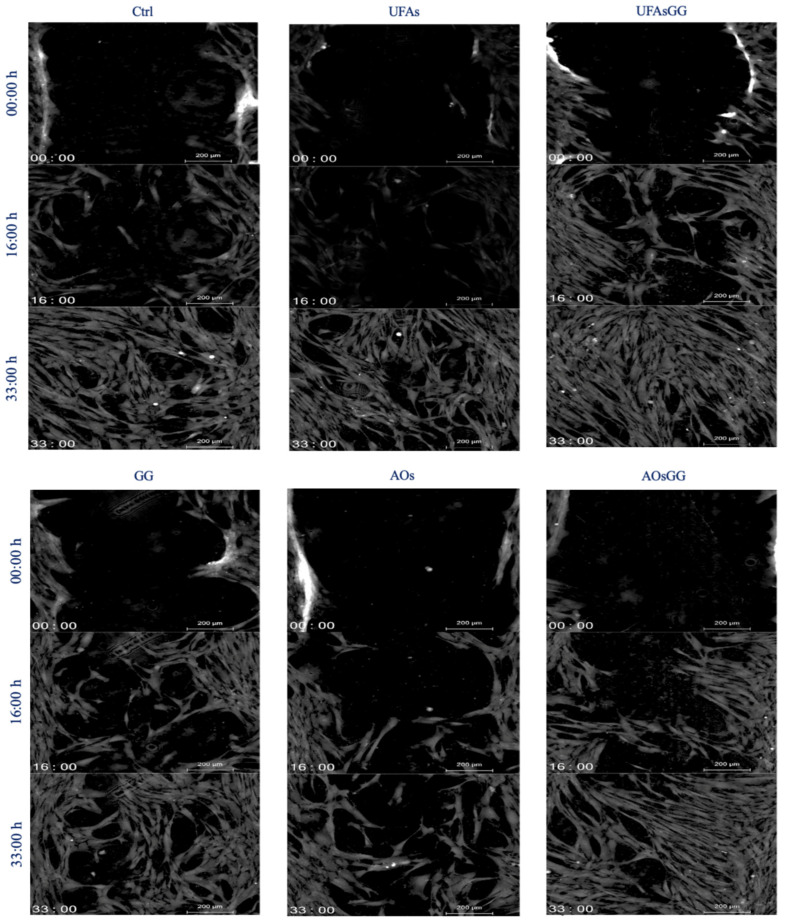
Quantitative phase imaging of fibroblasts at various times (0, 16, 33 h). Each image is representative of a scratch assay of three experimental groups.

Cell proliferation and cell migration are two essential processes that occur during wound healing. After assessing the biocompatibility of the prepared formulations associated with the regular proliferation of fibroblasts, the study continued by evaluating cell migration. The wound healing assay, one of the most widely used methods to assess the collective migration of cells, was then performed. Human fibroblasts were grown in a conditioned medium, enriched with components released by Spanish broom gauze, for 48 h. The results of the scratch test are shown in Figure 10. The fastest closure due to the nanosystems is clearly evident. After 33 h of treatment, the *GG* root extract-loaded UFAs lead to a complete closure of the simulated wound, whereas the other samples take longer.

**Figure 11:**
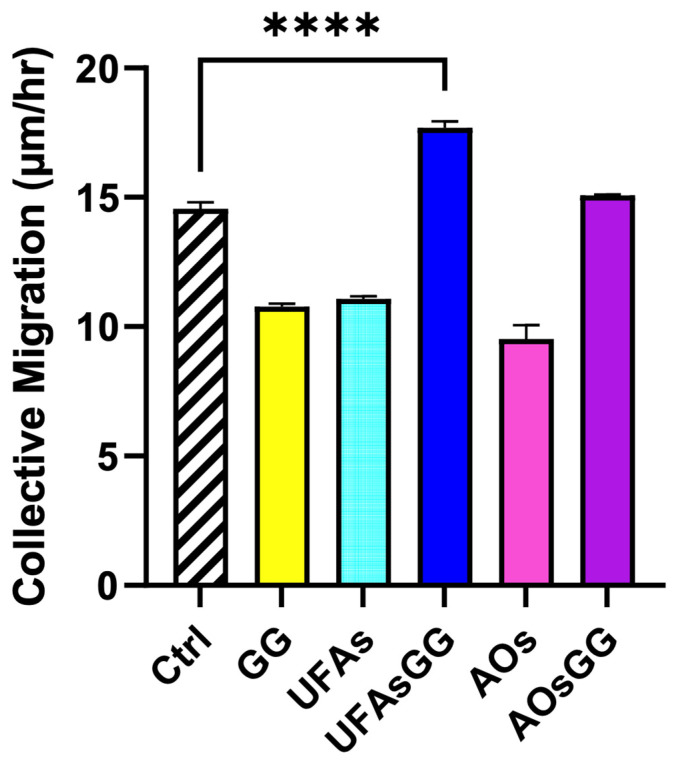
Collective migration of fibroblasts treated with GG root extract and unloaded and loaded nanovesicles. Data are expressed as the mean ± SD, n = 3 (**** *p* < 0.0001).

Comparing the two nanovesicles, it is worth noting that the GG root extract-loaded AOs, similar to the GG root extract-loaded UFAs, showed the ability to promote cell migration, but to a lesser extent. This finding is confirmed by evaluating the collective migration rate using the innovative Livecyte instrument, which utilizes ptychography to capture relative phase shift information (Figure 11).

## 3. Materials and Methods

### 3.1. Materials

The roots of *Glycyrrhiza glabra* L. were provided by Caffo 1915 Group Srl (Limbadi, Vibo Valentia, Italy), and vouchers of the dried plant material were deposited in the Department of Pharmacy and Biotechnology, University of Bologna (Via San Donato 19/2, Bologna, Italy). Oleic acid was purchased from FarmaLabor (Canosa di Puglia, Italy). Lipoid H90 (fat-free sunflower lecithin with 90% phosphatidylcholine, non-GMO) was sourced from Lipoid HmbH (Ludwigshafen, Germany). Ethanol and methanol (purity > 99.8%) and all the solvents were purchased from Sigma-Aldrich (Milan, Italy), as well as 2,2-diphenyl-1- picrylhydrazyl radical (DPPH), calcium carbonate, and linoleic acid (purity >97%). Folin-Ciocalteu reagent was sourced from Titolchimica (Pontecchio Polesine, Italy). Spanish broom dressings were provided by Professor Giuseppe Chidichimo of the University of Calabria (Arcavacata di Rende, CS, Italy). Dulbecco’s modified Eagle medium supplemented with 4.5 g/L of D-glucose was purchased from Sigma-Aldrich Co. (St. Louis, MO, USA), as well as 3-(4,5-Dimethylthiazol-2-yl)-2,5-Diphenyltetrazolium Bromide (MTT) reagent. Fetal bovine serum (FBS), trypsin/EDTA (5 g porcine trypsin and 2 g EDTA), L-glutamine, penicillin, and streptomycin were purchased from Euroclone S.p.A. (Milan, Italy). WS1 human fibroblasts were sourced from ATCC (American Type Culture Collection ATCC, Manassas, VA, USA). Tryton X100 was sourced from Sigma-Aldrich (Milan, Italy); Tert-butyl hydroperoxide (TBH) was purchased from Acros-organics (Geel, Belgium); and 2HDCF-DA was purchased from Sigma-Aldrich (Milan, Italy).

### 3.2. Preparation of GG Root Extract

In total, 15 g of shredded roots was extracted through maceration in the dark for 72 h using 300 mL of H_2_O:EtOH 50:50 *v*/*v.* According to Um et al. [20], this solution is able to extract the highest amount of antioxidant and bioactive compounds. Moreover, ethanol has the ability to extract phenolics and flavonoids, while water facilitates the extraction of phenolic acids and promotes the swelling of the vegetal matrix [20]. Subsequently, the hydroalcoholic extract was filtered through paper filters, and the remaining powder was extracted twice using 150 mL of the extracting phase, with the powder being shaken for 1 h each time and then filtered. All the extracts were reunited, and then the solvents were evaporated first through rotavapor (Buchi R-300, Buchi Italia s.r.l., Cornaredo (MI), Italy), which removed ethanol. The extract was then frozen at −20 °C for 1 day. Subsequently, it was placed in the freeze-dryer (Christ, Milan, Italy) for 48 h, which ensured the complete removal of water.

### 3.3. GG Root Extract Phenolic Richness Determined by HPLC

The phenolic richness of the GG root extract was estimated based on its GZ content. To this aim, an HPLC-DAD method was used [21]. The instrumentation was a JASCO PU-2089 quaternary pump, a Jasco UV/Vis MD 910 PDA detector, and an autosampler Jasco AS-2057 (Jasco, Tokyo, Japan). The analysis was carried out at 35 °C on a Poroshell 120, 2.7 mm (4.6 × 150 mm) C18 column (Agilent Technologies, Santa Clara, CA, USA) with a flow rate of 0.8 mL/min. Elution started with 98% eluent A (2% acetic acid in HPLC-grade water) and 2% eluent B (2% acetic acid in HPLC-grade acetonitrile). Then, the gradient was 95% A in 10 min., to 90% A in 7 min., to 83% A in 6 min., to 70% A in 6 min., to 0% A in 6 min., to 98% A in 2 min. Identification and quantification were accomplished by using standard solutions of GZ (Sigma Aldrich, Milano) at concentrations spanning from 1 mg/L to 240 mg/L and taking into account the UV/VIS spectrum and the retention time of the peak. GZ eluted at 34.95 min and was quantified (mg/mg extract) via the external standard method. Before the analysis, the extracts were diluted 1:4 with eluent A and filtered using PDVF syringe filters (0.45 mm). Analyses were carried out in triplicate.

### 3.4. Total Phenolic Content, Total Flavonoid Content, and Antioxidant Activity

The total phenolic content (TPC) was determined using the Folin–Ciocalteu reagent. Briefly, 0.2 mL of an aqueous extract solution (1 mg/mL) was added to 1 mL of 1:10 diluted Folin–Ciocalteu’s phenol reagent, followed by the addition of 0.8 mL of sodium carbonate solution (7.5% *w*/*v*). After 30 min in the dark at 40 ± 1.0 °C, the absorbance at 750 nm was measured using a UV-Vis 1601 spectrophotometer (Shimadzu, Milan, Italy). Distilled water served as a blank. The TPC was estimated from a standard curve of gallic acid (R^2^ = 0.998). All measurements were performed in triplicate, and the results were expressed as the gallic acid equivalent in µg/mg of *Glycyrrhiza glabra* extract (µgGAE/mg extract). The total flavonoid content (TFC) was determined using the pharmacopeial method, with minor modifications [6]. Briefly, 0.1 mL of 5% (*w*/*v*) AlCl_3_ solution was added to 0.9 mL of the extract solution (1 mg/mL *w*/*v*). After incubation at room temperature for 30 min in the dark, the absorbance was measured at 430 nm. The TFC was estimated from a standard curve of quercetin (R^2^ = 0.9998) and was expressed as µg of the quercetin equivalent (µg QE)/mg of the extract. The measurements were performed in triplicate. The antioxidant activity (AA) was determined by the 2,20-di-phenyl-1-picrylhydrazyl radical (DPPH) reduction assay, as described by Brand-Williams et al. (1995) [22], with minor modification. Briefly, a solution (1 mg/mL *w*/*v*) of the extract as well as ascorbic acid (used as a standard antioxidant compound) was mixed 1:1 with a solution of DPPH (0.1 mM in methanol) at room temperature. The mixtures were kept in the dark for 30 min, and the absorbance was measured at 517 nm. Methanol was used as a blank solution, and DPPH solution was used as the control. The test was carried out in triplicate. The results are expressed as a percentage of inhibition of the DPPH radical according to the following equation: Inhibition% = [(A0 − A)/A0], where A0 was the absorbance of the DPPH control and A was the absorbance of the sample with DPPH.

### 3.5. Antibacterial Activity of GG Root Extract

The antibacterial activity of the GG root extract was initially evaluated using the agar well diffusion assay, in accordance with the methodology outlined by Sallustio et al. (2024) [11]. In summary, 50 μL of each serial twofold dilution (ranging from 16.0 to 0.5 mg/mL) was spotted onto aseptically made wells (diameter 6 m) on Tryptone Soy Agar (TSA, Oxoid) medium. The medium had previously been seeded with approximately 10^6^ CFU/mL of the target microorganisms, namely, *Escherichia* (*E*.) *coli* DSM11250 (=ATCC 10536), *Enterococcus* (*E*.) *hirae* DSM3320 (=ATCC 10541), and *Staphylococcus* (*S*.) *aureus* DSM799 (=ATCC 6538). After a 24 h incubation period at 37 °C, the appearance of a clear halo surrounding the wells was related to the antimicrobial effect.

Strains sensitive to GG root extract by the agar well diffusion test were subject to the time–kill assay [11]. Tryptone Soy Broth (TSB, Oxoid) medium supplemented with GG root extract at different concentrations (16, 8, 4, 2, and 1 mg/mL) was inoculated with indicators at around 10^6^ CFU/mL. To discriminate whether the effect was bactericidal or bacteriostatic, population levels were assessed at time 0 as well as after 24 and 48 h of incubation at 37 °C by counting on TSA. Every test was carried out in triplicate.

### 3.6. Encapsulation of GG Root Extract

#### 3.6.1. Preparation of UFAs

GG root extract-loaded UFAs were prepared by solubilizing LipoidH90 (15 mg/mL) in the oily phase composed of oleic acid (30 mg/mL) and linoleic acid (10 mg/mL) at 32 ± 1 °C until complete solubilization. Subsequently, the lyophilized extract of *Glycyrrhiza glabra* L. (6 mg/mL) was solubilized in ultrapure water, and it was added drop by drop to the oily phase using a 5 mL syringe. The two phases were homogenized with a high-speed homogenizer (Ultra-Turrax T25 Basic, IKA-WERKE, Germany) for 2 min at 9500 rpm. Finally, ultrasonication with a probe (Ultrasonic convertor model n °C L4, serial C8087, Bergamo, Italy) for 5 min, in a bath of ice, was performed. The unloaded UFAs were prepared as the control.

#### 3.6.2. Preparation of AOs

GG root extract-loaded AOs were prepared by solubilizing LipoidH90 (15 mg/mL) in oleic acid (40 mg/mL) at 32 ± 1 °C until complete solubilization. Afterwards, the lyophilized extract of *Glycyrrhiza glabra* L. (6 mg/mL) was solubilized in ultrapure water, and it was added, drop by drop, to the oily phase using a 5 mL syringe. Two phases were homogenized with a high-speed homogenizer (Ultra-Turrax T25 basic IKA_WERKE) for 2 min at 9500 rpm. Finally, ultrasonication with a probe (Ultrasonic convertor model n°CL4, serial C8087) for 5 min, in a bath of ice, was performed. The unloaded AOs were prepared as the control.

#### 3.6.3. Characterization of Vesicles

##### Size, PDI, and Zeta Potential Measurement

The prepared nanovesicular systems were characterized by their vesicle size and their polydispersity index (PDI). The UFAs and AOs were diluted (1:1000 *v*/*v*) in MilliQ water before the analysis. The size and PDI were measured through Dynamic Light Scattering (90Plus Particle Size Analyzer, Brookhaven Instruments Corp., Holtsville, NY, USA), while the zeta potential measurements were carried out at 25 °C through a Nicomp™ 380 ZLS instrument (Menlo Park, CA, USA) after the same dilution (1:1000 *v*/*v*).

##### EE

To evaluate the amount of GZ encapsulated in the vesicles, the entrapment efficiency (EE) was determined through the dialysis method. Specifically, 1 mL was purified from the non-incorporated components through dialysis (Spectra/Por^®^ membranes: 14 kDa MW cut-off) in water (0.5 L) for 2 h at room temperature, with the distilled water refreshed after 30 min (2 L in total). At the end of the purification process, the GZ content, before and after dialysis, was estimated through HPLC analyses, as described in paragraph 3.3. The nanovesicles were previously treated with a methanol solution (1:4 ratio); afterwards, the EE was calculated as a percentage of GZ after dialysis versus that before dialysis, as in the following formula:

EE% = (GZ% dialyzed sample/GZ% non-dialyzed sample) × 100

##### Stability Studies

The physical stability of UFAs and AOs was assessed by monitoring the diameter of the nanovesicles and the polydispersity every 7 days for 4 weeks, and then every 30 days for the next 6 months, through Dynamic Light Scattering (DLS) (90Plus Particle Size Analyzer, BTC). All vesicle suspensions were stored in a refrigerator at 4.0 ± 1.0 °C in the dark. For this study, before the measurement of the size and PDI, aliquots of vesicle suspensions were diluted in ultrapure water (1:1000; *v*/*v*) at predetermined periods (0, 7, 14, 21, 28, 60, 90, 120, and 150 days).

The accelerated stability of the loaded and unloaded vesicles was performed with the multi-wavelength analytical photocentrifuge LUMiSizer^®^ (L.U.M. GmbH, Berlin, Germany) using STEP-Technology^®^. A volume of about 1 mL of nanovesicular systems was placed in a specific cuvette (PA110-135XX), subjected to increasing rotor speeds up to 4000 rpm. The evolution of the transmission profiles, trace instability phenomena, and instability indices were analyzed using SEPView^®^ software 6.4.678.6069.

#### 3.6.4. In Vitro Release Studies

The GZ release profiles from lipid vesicles were carried out using a Franz-type static glass diffusion cell (15 mm jacketed cell with a flat-ground joint and clear glass with a 12 mL receptor volume; diffusion surface area = 1.77 cm^2^) equipped with a V6A Stirrer (PermeGearInc., Hellertown, PA, USA). Spectra/Por^®^ membrane (14 kDa MW cut-off) was placed between the receptor and the donor compartments, and 12 mL of a mixture of 3:7 (*v*/*v*) ethanol/pH 7.4 buffer was used as the receptor medium. The donor compartment was filled with 1 mL of the vesicle suspension. The systems were kept at 32.0 ± 1.0 °C under magnetic stirring (100 rpm/min). At predetermined time points, the samples (0.2 mL) were withdrawn, and the amount of GZ in each aliquot was analyzed by HPLC (as reported in Section 3.3). The GZ release profiles were performed in triplicate.

#### 3.6.5. Cell Viability Studies

The human fibroblast WS1 cells were grown in DMEM high glucose, supplemented with 10% fetal bovine serum, 2 mM L-Glutamine, 100 units/mL penicillin, and 100 µg/mL streptomycin, at 37 °C in a 5% CO_2_/95% air humidified atmosphere. To assess the biocompatibility of the nanovesicular systems, an MTT assay was performed. Briefly, WS1 cells were seeded at 7000 cells/well in a 96-well plate and treated for 24 and 48 h with different concentrations of UFAs or AOs (0.15% and 0.31% *v*/*v*) or pure extract (same dilution of nanovesicles, 9.3 μg/mL and 18.6 μg/mL). Subsequently, 10 µL of MTT solution (5 mg/mL) was added, and after 4 h of incubation, the media were replaced with 100 µL of isopropanol to solubilize the formazan crystal. Absorbance at 570 nm was measured by means of the multimode plate reader EnSpire (PerkinElmer, Waltham, MA, USA).

#### 3.6.6. Cell Cycle Evaluation by Flow Cytometry

Cell cycle analysis was performed according to Nüsse et al. [23]. Briefly, 2 × 10^6^ cells were centrifuged, and 1 mL of solution I (0.584 g/L NaCl, 1.139 g/L sodium citrate, 10 mg/L RNase, 0.3 mL/L Nonidet P-40) was added to the cell pellet. After 30 min, 1 mL of solution II (100 mg/L propidium iodide (PI), 0.25 M sucrose, 15 g/L citric acid) was added to complete the fixing and staining. The cell suspension was mixed and kept at 4 °C until flow cytometry measurement. Flow cytometric analyses were carried out using a S3 Cell Sorter (Bio-Rad, Hercules, CA, USA) equipped with an Argon laser. Finally, the data were analyzed with FCSalyzer software.

#### 3.6.7. Biosafety

Human blood was provided according to ethical standards, the Declaration of Helsinki, and national and international guidelines from the Immunohematology and Transfusion Medicine Service Bologna Metropolitan Area (Protocol number 0000816 of 23/02/2024). Erythrocytes from human blood were collected by centrifugation at 116 g for 15 min and then diluted to 5% (*v*/*v*) with PBS. Briefly, a biological sample (2 mL) was prepared by adding 1 mL of GG root extract (37.2 μg/mL and 18.6 μg/mL), loaded and unloaded UFAs, or AOs diluted in PBS (0.62% and 0.31% *v*/*v*) to 1 mL (5%) red blood cell (RBC) suspension in isotonic buffer solution (pH 7.4). Tryton × 100 (0.1% *v*/*v*) and phosphate buffer solution were used as positive and negative controls, respectively. The samples were incubated for 60 min at 37 °C and then were centrifuged at 1100× *g* for 3 min. The absorbance of the supernatant was detected at 542 nm. The Hemolysis rate was calculated according to the following equation:

- Hemolysis% = [(Abs sample − Abs negative control)/(Abs positive control − Abs negative control)] × 100

#### 3.6.8. Intracellular ROS Assay

Reactive oxygen species were detected in intact cells, according to Oparka et al. [24], with minor modifications. Briefly, WS1 fibroblasts were seeded at a density of 30,000 cell/cm^2^ in a 96-well plate and incubated for 24 h to allow for adhesion. To induce oxidative stress, the cells were exposed to 150 μM tert-butyl hydroperoxide (TBH) in PBS for 30 min. Then, the cells were treated for 24 h with GG root extract (18.6 µg/mL), loaded and unloaded UFAs, and AOs (0.31% *v*/*v*). After the treatment, the cells were incubated with 2 μM H_2_DCFDA (Thermo Fisher Scientific, Waltham, MA, USA) for 30 min. After washing, the samples were analyzed under fluorescence microscopy using a Nikon Eclipse TE300 confocal microscope, applying a λ_exc_ of 488 nm and a λ_em_ of 515 nm. Finally, the fluorescence intensity of the acquired images was measured using FIJI software [25].

### 3.7. Preparation of Spanish Broom Wound Dressing

#### 3.7.1. Impregnation of Wound Dressing with Vesicles

The final wound dressing was prepared using Spanish broom as the supporting material. Specifically, 100 μL of prepared vesicles (as reported in Section 3.6.1 and Section 3.6.2), diluted in 0.31% *v*/*v* PBS, were absorbed onto a 1 × 1 cm^2^ piece of Spanish broom for one hour. Then, the dressings were placed in 3 mL of the culture medium and kept for six hours at 32 °C under shaking at 200 rpm. Next, the dressings were removed and the media was centrifuged at 2000 rpm for 10 min and filtered with a 0.45 μm syringe filter. Finally, the conditioned medium was supplemented with 10% fetal bovine serum, 2 mM L-Glutamine, 100 units/mL penicillin, and 100 µg/mL streptomycin.

#### 3.7.2. Wound Healing Assay

The wound healing capacity of the UFAs and AOs was evaluated by a scratch test. The WS1 fibroblasts were seeded in a 24-well plate at a density of 65,000 cells per well and incubated until confluence. The cell monolayers were manually scraped with a p200 pipette tip and then washed twice with PBS to remove cell debris. Next, WS1 fibroblasts were treated with conditioned media (as reported in Section 3.6.1) with GG root extract, GG root extract-loaded UFAs, GG root extract-loaded AOs, UFAs, and AOs. QPI (quantitative phase imaging) was executed with the Lifecyte^TM^ imaging system (Phase Focus, Sheffield, UK), and images were acquired every 1 h for 48 h. Finally, the data were analyzed with the Cell Analysis Toolbox software (Phase Focus, Sheffield, UK).

### 3.8. Statistical Analysis

Every experiment was performed three times, and all results are shown as the mean ± standard deviation (SD). The data from all the experiments were analyzed using the *t*-test or one-way and two-way ANOVA tests. The GraphPad Prism software, version 8.0 for Windows (GraphPad Software, San Diego, CA, USA), was used for all graphs, computations, and statistical analyses.

## 4. Conclusions

In this work, new nanovesicles based on unsaturated fatty acids were successfully used to encapsulate *Glycyrrhiza glabra* extract. Thanks to their suitable physico-chemical properties, *Glycyrrhiza glabra* extract-loaded UFAs and AOs are good candidates for developing new pharmaceutical products for wound care. In fact, biological studies have demonstrated their biosafety and antioxidant properties, two important requirements for rapid wound closure, avoiding medical consequences. Considering these promising characteristics, we developed a new tool for wound healing made of Spanish broom dressing impregnated with nanoformulations. Additionally, the scratch test demonstrated that Spanish broom dressing impregnated with GG root extract-loaded UFAs accelerated skin wound closure consistently, indicating the efficacy of this innovative medical device for the treatment of wounds. Future studies will focus on the in vivo evaluation of the new wound dressing developed in this work.

## Figures and Tables

**Table 1:** Physico-chemical characterization of nanovescicles.

	Size (nm)	PDI	ζ Potential	EE%
Unloaded UFAs	219.06 ± 19.88	0.223 ± 0.028	−30.24 ± 2.75	-
UFAsGG	158.96 ± 3.60	0.168 ± 0.021	−30.16 ± 1.37	64.91 ± 3.27
Unloaded AOs	211.02 ± 7.40	0.217 ± 0.037	−26.63 ± 5.16	-
AOsGG	158.19 ± 3.54	0.207 ± 0.039	−28.65 ± 2.85	67.61 ± 5.94

## Data Availability

The data are contained within the article.

## Supplementary Materials

The following supporting information can be downloaded at: https://www.mdpi.com/article/10.3390/molecules29163811/s1, Figure S1: Growth inhibition in the presence of different concentrations of GG root extract
