# Supplementary material for: A New Wound-Healing Tool Based on Glycyrrhiza glabra Extract-Loaded Ufasomes on Spanish Broom Dressings
Source: Molecules. 2024 Aug 11;29(16):3811. doi: 10.3390/molecules29163811 (PMC11357027; doi:10.3390/molecules29163811)

Supplementary Figure S1

Agar well diffusion assays of the of the GG root extract (from 16.0 to 0.5 mg/mL) against *Staphylococcus* (*S.*) *aureus* DSM799 (=ATCC 6538) and *Enterococcus* (*E.*) *hirae* DSM3320 (=ATCC 10541).

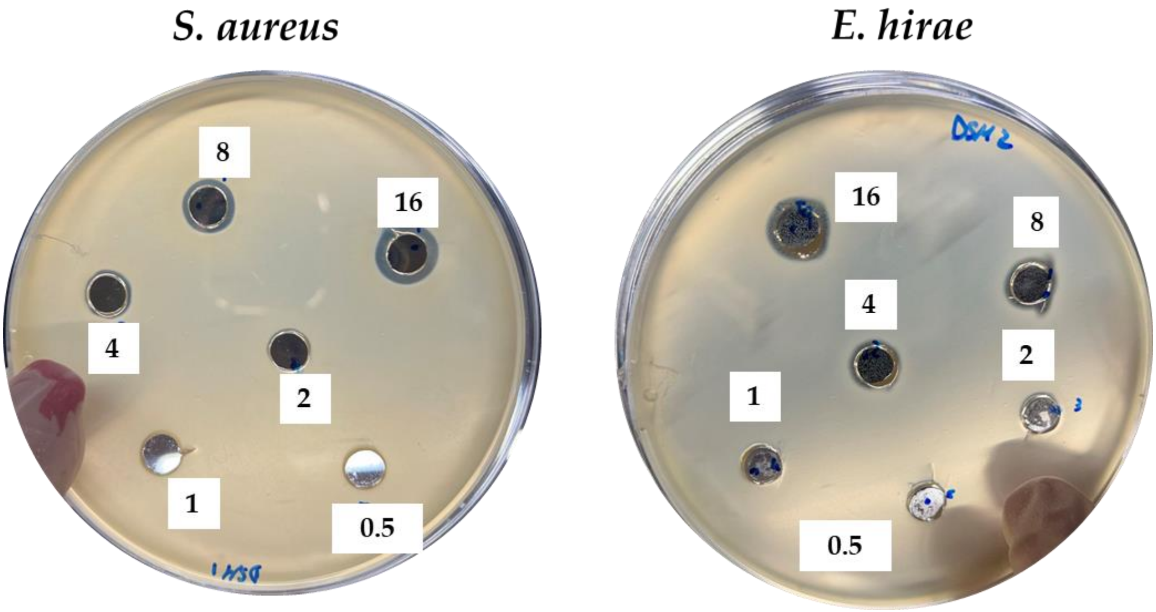

Supplement: Supplementary file 1 [file molecules-29-03811-s001.zip › molecules-3088487-supplementary.pdf]
